# Editorial to “Acute occlusion of the left main coronary artery following impedance rise after high‐frequency catheter ablation”

**DOI:** 10.1002/joa3.13136

**Published:** 2024-08-20

**Authors:** Wei‐Ta Chen

**Affiliations:** ^1^ Division of Cardiology, Department of Internal Medicine Taipei Medical University Hospital Taipei Taiwan; ^2^ Department of Internal Medicine, College of Medicine, School of Medicine Taipei Medical University Taipei Taiwan

**Keywords:** coronary artery, impedance, radiofrequency ablation

In this issue, Takafumi Koyama presented a case of frequent premature ventricular complexes (PVC).^1^ The authors performed radiofrequency ablation (RFA) for the PVCs near the left coronary cusp with an irrigated ablation catheter. The catheter impedance increased suddenly during ablation and the patient encountered acute left coronary artery occlusion. The situation was solved by coronary artery stenting and repeated balloon dilation.

Catheter impedance is a dynamic value that reflects the electrical resistance between the catheter tip and the surrounding tissue. As radiofrequency energy is delivered, the tissue adjacent to the catheter tip heats up. This heat causes changes in the tissue's electrical properties, leading to a decrease in impedance.^2^ A gradual decrease in impedance typically indicates effective tissue heating and lesion formation. However, abrupt changes in impedance can indicate several clinical situations. Steam pop, catheter movement, catheter fracture, tissue charring, and entry into a small vessel are conditions with sudden increase in catheter impedance. On the contrary, catheter tip erosion and tissue penetration may lead to a sudden drop in catheter impedance.

The type of catheter used, irrigated or nonirrigated, can also influence impedance measurements. Nonirrigated catheters rely solely on the conductive properties of the tissue for heat dissipation. As the tissue heats up, impedance tends to decrease more rapidly with irrigated catheters. There is a higher risk of steam pops and tissue damage due to the lack of cooling. For the irrigated catheters, the continuous saline delivery cools the area and improves heat dissipation. This results in a slower rate of impedance decrease. The risk of steam pops and tissue damage is reduced because of the cooling effect. While irrigated catheters generally provide better control over tissue temperature, impedance monitoring remains crucial for both types of catheters to optimize ablation and prevent complications.

In the presented case, the RFA was performed in the aorta near the left coronary artery ostium. During RFA, the catheter impedance once suddenly increased. The condition may indicate a catheter moving from the aorta into the coronary artery. Coronary arteries have a significantly lower blood flow rate compared with the aorta. This change in blood flow directly impacts the catheter's electrical environment. The narrower diameter of the coronary artery also leads to a smaller contact area between the catheter and the vessel wall. This reduced contact area alters the electrical properties of the system. The tissue composition of coronary arteries differs from that of the aorta, further influencing the electrical conductivity.^3^

When performing RFA near the coronary cusps, the rapid impedance increase serves as a strong indicator that the catheter has entered a coronary artery, which is a critical event during ablation procedures. It requires immediate attention from the physician to prevent complications such as coronary artery occlusion. In this case, although the authors stopped further RFA when noting the increase in impedance, left coronary occlusion was happened.

For the concerns of coronary artery injury during RFA near the coronary cusps, coronary angiography is strongly recommended before RFA.^4^ Coronary angiography provides a precise visualization of the coronary artery origins, their course, and their relationship to the target ablation site. Knowing that helps risk mitigation and procedural planning. In certain cases, with contraindications for contrast, such as poor renal function or allergic history, intracardiac echography or transesophageal echocardiography may also be a good alternative imaging modality.^5^

Ablation in the coronary cusps is a complex procedure requiring meticulous attention to detail. Given the proximity of the coronary ostia, coronary angiography before RFA is useful to accurately map their location and prevent accidental occlusion. Impedance monitoring, a crucial tool during ablation, can provide real‐time feedback on tissue interaction. However, it is important to note that impedance changes alone cannot definitively indicate coronary artery entry. A sudden increase in impedance might suggest catheter proximity to a vessel, but confirmation through other modalities, such as fluoroscopy and electrograms, is crucial. Ultimately, a combination of advanced imaging, careful catheter manipulation, and experienced operators is essential for successful and safe aortic cusp ablation.

## FUNDING INFORMATION

None.

## CONFLICT OF INTEREST STATEMENT

Authors declare no conflict of interests for this article.

## ETHICS APPROVAL STATEMENT

None.

## Data Availability

None.
